# Feedback within the Inter-Cellular Communication and Tumorigenesis in Carcinomas

**DOI:** 10.1371/journal.pone.0036719

**Published:** 2012-05-17

**Authors:** Felix Rückert, Robert Grützmann, Christian Pilarsky

**Affiliations:** Department of General, Thoracic and Vascular Surgery, University Hospital Carl Gustav Carus, Dresden, Germany; Hungarian Academy of Sciences, Hungary

## Abstract

The classical somatic mutation theory (SMT) of carcinogenesis and metastasis postulates that malignant transformation occurs in cells that accumulate a sufficient amount of mutations in the appropriate oncogenes and/or tumor suppressor genes. These mutations result in cell-autonomous activation of the mutated cell and a growth advantage relative to neighboring cells. However, the SMT cannot completely explain many characteristics of carcinomas. Contrary to the cell-centered view of the SMT with respect to carcinogenesis, recent research has revealed evidence that the tumor microenvironment plays a role in carcinogenesis as well. In this review, we present a new model that accommodates the role of the tumor microenvironment in carcinogenesis and complements the classical SMT. Our “feedback” model emphasizes the role of an altered spatiotemporal communication between epithelial and stromal cells during carcinogenesis: a dysfunctional intracellular signaling in tumorigenic epithelial cells leads to inappropriate cellular responses to stimuli from associated stromal or inflammatory cells. Thus, a positive feedback loop of the information flow between parenchymal and stromal cells results. This constant communication between the stromal cells and the tumor cells causes a perpetually activated state of tumor cells analogous to resonance disaster.

## Introduction

### Current Concepts on Carcinogenesis

**Figure 1:**
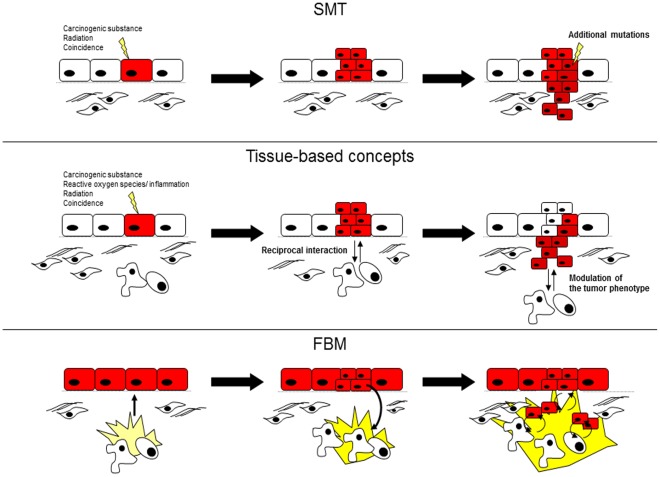
Different theories of carcinogenesis. The SMT postulates that mutations in oncogenes lead to cell-autonomous growth. Additional mutations are necessary for invasive growth (**A**). The tissue-based models assume that reciprocal communication between tumor and stromal cells can influence the phenotype of the tumor cells. The tissue-based models question the autonomy of the mutations in epithelial cells and the sovereignty of the tumor cells in determining the cancer phenotype (**B**). In the FBM, mutations in genes could show a normal phenotype under physiological conditions but can be activated by inflammation. Under inflammatory conditions, tumor and stromal cells interact to form a positive feedback loop. Additional stromal and inflammatory cells become attracted to the inflammatory micromilieu (**C**).

One of the current prevailing theories of carcinogenesis is the somatic mutation theory (SMT) of carcinogenesis and metastasis, which postulates that cancer is a disease based on the transformation of individual cells. SMT proposes that mutations in tumor suppressor genes and oncogenes lead to the uncontrolled proliferation of tumor cells in a cell-autonomous fashion. Tumors progress to more malignant stages of disease by further accumulating mutations in a multistep process [1], [2]. In the SMT, cells of the tumor microenvironment play a simple, subservient role to that of the original mutated cell (**Figure 1**) [3].

SMT has been criticized because the accumulation of the three to six mutations that are necessary for a cell to become malignant might not be possible in the normal life span of a single cell [2], [4], [5]. Another very important criticism of SMT is that the malignant phenotype of epithelial cancer cells seems to be reversible. Several studies have shown that isolated parenchymal cells from neoplastic tissues lose their tumorigenic phenotype when transplanted into normal tissues [6], [7], [8].

Carcinomas are heterogeneous and structurally complex tumors, and more credence has recently been given to additional cell types that contribute to the carcinogenesis and pathophysiological properties of tumors [9], [10], [11], [12]. This perception has led to newer, tissue-based theories of carcinogenesis [7], [9], which postulate that transformed cells are not autonomous but can be affected by reciprocal interaction between the parenchymal and stromal cells [7] (**Figure 1**).

A study published by *Bissell et al.* showed that cancer could be considered as a breakdown in communication between the epithelium and the surrounding stroma. Transformed cells could send inappropriate signals to stromal cells that could lead to aberrant responses that facilitate tumorigenesis. Defects in tumor-stroma paracrine signaling lead to increasingly aberrant cellular behavior and ultimately result in increased cellular complexity and heterozygosity. Likewise, alterations in intercellular communication could precede and cause the development of carcinomas because chronic exposure to DNA-damaging agents induces malignant transformation [9]. According to the results from a study performed by *Axelrod et al*., interactions between tumor cells and stromal cells could be necessary for carcinogenesis. This study indicated that subclones within a tumor require an extended period of time to accumulate a tumorigenic complement of mutations. During this the process of transformation, tumor cells could depend on stromal interactions for support and growth [13]. Therefore, tissue-based theories question the autonomy of mutations in epithelial cells and the sovereignty of tumor cells in acquiring a cancerous phenotype. In this review, we present the feedback model (FBM) as a novel tissue-based model. The FBM is based on the assumption that certain mutations do not drive proliferation and activation in cancer cells in a cell-autonomous process, but rather in a cell-heteronomous fashion by passively enabling the deregulation of intracellular signaling processes within tumor cells. Dysfunctional intracellular signaling leads to an aberrant response to extracellular stimuli resulting in a positive feedback loop in the information flow between tumor cells and stromal cells. Feedback between the tumor and the stroma could perpetuate proliferative or inflammatory states within the tumor micromilieu.

In contrast to the SMT, our feedback model postulates a cell-heteronomous action of certain mutations in the malignant transformation of epithelial cells. In the FBM, malignant transformation is dependent on inflammatory changes in the tumor-stroma micromilieu. The FBM is a tissue-based model of carcinogenesis because it assumes that communication between stromal cells and tumor cells is important for tumorigenesis. However, the FBM is unique from typical tissue-based models because the FBM assumes that different interactions between different cell types result in a positive feedback loop to encourage tumor growth.

To illustrate the FBM, we will use pancreatic ductal adenocarcinoma (PDAC) as an example. One of the main goals of this review is to argue that the FBM is completely consistent with the current body of cancer research. This review will also explore the implications of the FBM on the different pathophysiological features of carcinomas.

## Results

### Feedback of Intercellular Signaling: Basic Assumptions

Stromal cells and their secreted products influence adjacent epithelial cells through transient signaling to elicit specific cellular reactions in the target cells. Physiologically, the effects in the target cells reciprocally produce stimuli for neighboring cells [14].

All signals from the environment are integrated by the intrinsic cellular information processing mechanism, which has three important functions: (i) to transfer and magnify extracellular stimuli to induce cellular response (vertical processing), (ii) to integrate different extrinsic and intrinsic stimuli and relay the signal to other cells (horizontal processing) and (iii) to timely restrict the cellular answer.

Vertical signal processing occurs by receptor internalization, signal enhancement by adaptor proteins and positive or negative feedback. Horizontal signal processing is achieved by branching from signal integration nodes thereby interconnecting different signaling pathways. The time course of the cellular response to stimuli is determined by positive and negative feedback loops within the signaling pathways [15]. In biological systems, negative feedback systems are an integrative part of each signaling pathway. Negative feedback allows each cellular reaction to be terminated when sufficient response or product is produced providing a way for the cell to control signaling pathways and target gene expression [15]. The mechanisms by which signaling pathways are regulated are genetically encoded. Therefore, in the absence of mutations or disorders in the activation of regulatory genes, cells should not respond aberrantly to stimuli from neighboring cells.

The FBM assumes that mutations in genes involved in intracellular signal processing could hinder the ability of the cell to accurately respond to external stimuli. This could result in a dysfunction in the finely tuned intercommunication between cells. Mutations in genes such as these could lead to a disproportionately high level of activation of the respective signaling pathways, paradoxical activation or inactivation of signaling pathways or prolonged activation of certain signaling pathways.

Mutations in proteins involved in signal processing could theoretically manifest as a normal phenotype under physiological conditions. However, during inflammatory or regenerative processes, several different cell types secrete many different signaling molecules into the extracellular compartment. Confronted with an abundance of signaling molecules that may or may not be antagonistic, epithelial cells must rely on their own ability to appropriately process intracellular signals to adjust to the situation.

Considering the three main functions of the intracellular signal processing machinery, disproportionately high activation of inflammatory pathways of epithelial cells could result in the recruitment of additional inflammatory cells to the vicinity of the epithelial cells enhancing the inflammatory environment. Furthermore, paradoxical activation or inactivation of signaling pathways in the context of acute inflammation could elicit paradoxical reactions in neighboring stromal cells as well. Finally, the inability of epithelial cells to deactivate signaling pathways could also lead to a prolonged inflammatory micromilieu. The culmination of these dysfunctions could result in a sustained inflammatory environment or could induce the proliferation of epithelial cells. Under these circumstances, the stromal cells would continually become activated by the inflammatory environment forming a positive feedback loop of intercellular signaling.

### Prerequisites for the Feedback Loop Model

For a feedback loop in the communication between stromal cells and tumor cells to exist, two prerequisites are necessary: the existence of a permissive extracellular milieu and mutations in the corresponding intracellular signaling pathway in the epithelial cell. The appropriate mutation within the signaling pathway should occur in a component that is localized at an important node of the signal-processing pathway. There are numerous genes that fit this description, and we will refer to these genes as “loop genes”. A key requirement of the feedback loop model is that the stimuli found in the micromilieu must suit the intracellular defect. This is analogous to the mechanical principle of resonance disaster whereby a certain wave frequency can cause a resonance disaster only when it matches the resonance frequency of the system.

Under physiological conditions within the micromilieu, the mutation in the loop gene could be silent and confer a normal phenotype on the cell. However, once the positive feedback loop is initiated, secreted signaling factors can carry the information to neighboring epithelial cells resulting in an expansion of the feedback system. The FBM is consistent with the SMT because some oncogenes and tumor suppressor genes have pleiotropic functions, which, in addition to their classical role in cellular signaling, might act as loop genes.

### Example of the Feedback Loop Model in Pancreatic Cancer

In pancreatic cancer, KRAS mutations and NF-κB activation is observed in almost all cases [16], [17], [18], [19]. KRAS is a member of the small GTPase superfamily and is involved in many signal transduction pathways especially growth signaling pathways [20]. Although KRAS mutations tend to activate the signal transduction pathways, not all KRAS mutations result in cancer formation. Up to 39% of individuals without cancer were shown to carry mutations in this gene [21].

The NF-κB signaling pathway induces the expression of a large number of genes that have important functions in the regulation of immune and inflammatory responses including cytokines, chemokines, adhesion molecules and other immunoregulatory proteins. Additionally, canonical NF-κB controls the expression of proteins involved in proliferation [22], [23].

Physiologically, the proper regulation of NF-κB activation at epithelial interfaces is crucial for the maintenance of physiological tissue homeostasis. In inflamed pancreatic tissue, macrophages and other immune cells significantly stimulate NF-κB and cytokine expression in parenchymal cells [24], [25], [26], [27].

Normally, the activation of NF-κB in parenchymal cells is controlled by different negative feedback factors such as the deubiquitinating enzymes A20 and SOCS [28], [29]. If intact during the inflammatory conditions of the tumor micromilieu, these negative feedback factors should control the activation of epithelial cells in the vicinity.

**Figure 2:**
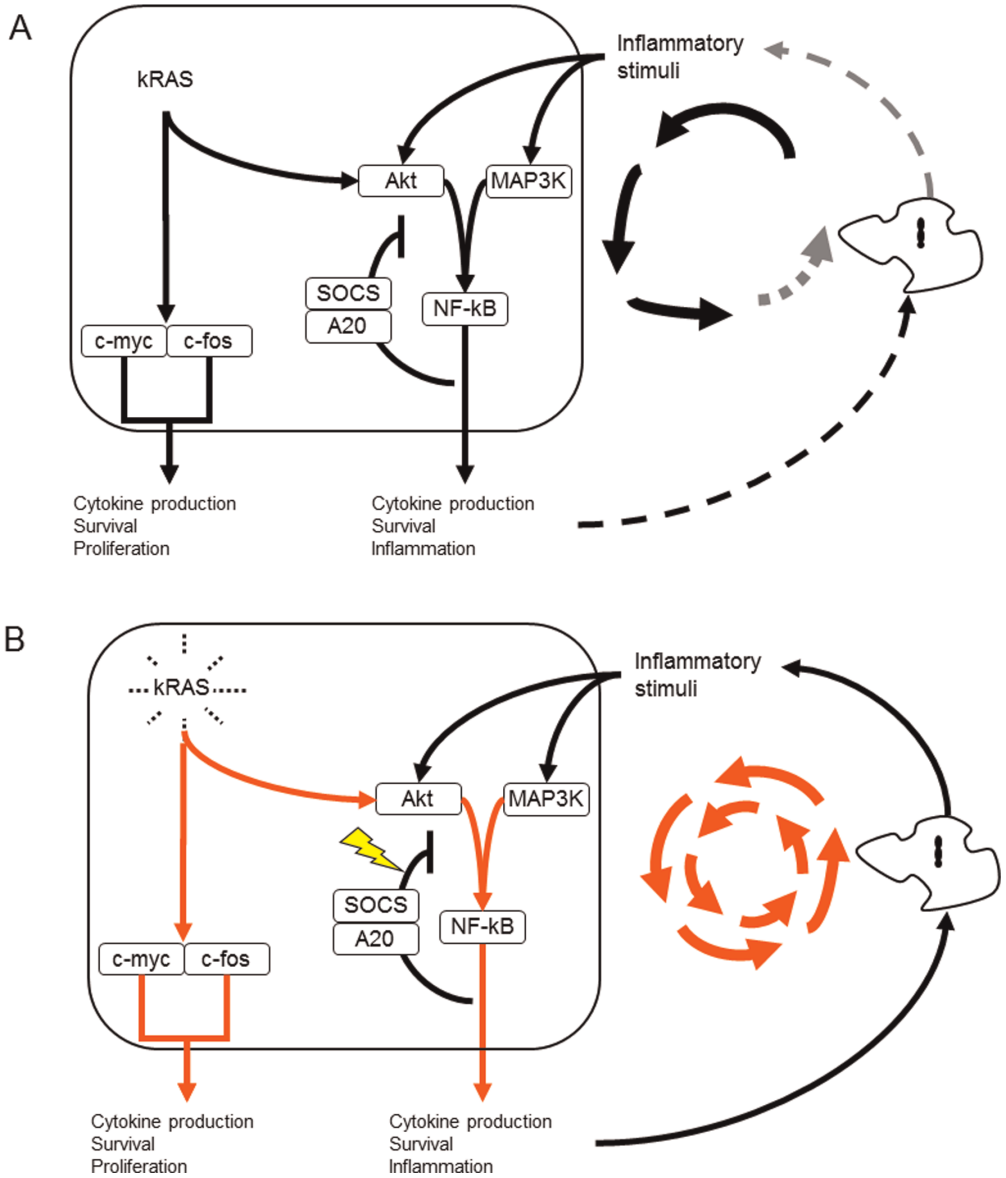
Normal activation of NF-κB and KRAS in an inflammatory environment. Negative feedback loop within the NF-κB pathway by SOCS and A20 counteracts the activated state of the epithelial cell (**A**). The non-physiological activation of KRAS impairs the negative feedback loop within the NF-κB pathway and leads to a perpetual inflammatory microenvironment (**B**).

However, KRAS has different interconnections with the NF-κB signaling such as the MAPK and Akt pathways (**Figure 2**) 30,31,32,33. In pancreatic cancer, interconnections between KRAS and the NF-κB signaling pathway have been previously described [34]. Activation of the NF-κB pathway by inflammatory conditions within the micromilieu could be enhanced by the prior activating KRAS mutations because KRAS mutation compromises the negative feedback mechanism of A20 and SOCS within the NF-κB pathway.

In this manner, the augmented activation of NF-κB and KRAS could largely potentiate the proliferative/inflammatory cellular response thereby prolonging the inflammatory state of the micromilieu. This could result in augmented cell division of the epithelial cells as well as the recruitment of macrophages and other mesenchymal cells to the microenvironment by inflammatory mediators forming a feedback of intracellular signaling. Therefore, KRAS could represent a loop gene.

## Discussion

### Similarities and Differences of the FBM to the Somatic Mutation Theory of Carcinogenesis and Metastasis

SMT considers cancer as a cumulative process of genetic instability and natural selection that ultimately leads to the malignant transformation of the cell [35]. Once transformed, the cell displays cell-autonomous growth. In this model, activation of the cell is static and irreversible (**Figure 1**). However, most of the identified mutations associated with cancer were found in fully developed tumors and thus might not explain the events that initiate carcinogenesis [7], [36].

The FBM suggests that the mutated genes could function in a different capacity than the classical oncogenes and tumor suppressor genes. The FBM suggests that intracellular mutations might act in a cell-heteronomous fashion. By supporting the communication between different cell types of the tumor, the mutations could lead to a perpetual inflammatory and/or proliferative micromilieu. Indeed, there could exist an overlap between the classical oncogenes or tumor suppressor genes and the putative loop genes because some of the classical genes could also initiate a feedback loop. Similarly to SMT, the FBM expects the occurrence of additional mutations because of the large number of proliferating cells within the tumor. In *in vitro* cell cultures, the accumulation of mutations could explain the fact that most tumor cell lines in culture can initially only grow in the presence of large amounts of growth factors and other hormones that mimic an inflammatory micromilieu [37]. However, some tumor cell lines can be adapted to growth in reduced serum or serum-free conditions [38]. Additional mutations could provide an autocrine feedback loop thereby rendering the cell lines independent from stromal cell support.

### Similarities and Differences of the FBM to the Tissue-based Models

The tumor stroma seems to have an important influence on carcinogenesis [6]. Tissue-based models of carcinogenesis postulate that the micromilieu and microarchitecture can modulate the tumor phenotype (**Figure 1**). Previous studies have provided examples for different conditions under which the stroma can positively or negatively modulate the phenotype of certain mutations and, therefore, the pathophysiology of the tumor [6], [39]. Tissue-based models question the dominance of the mutations in epithelial cells and the sovereignty of the tumor cells in determining the cancer phenotype. However, previous tissue-based models have proposed that malignant transformation is gained according to the SMT, and the molecular mechanisms, such as those that recruit stromal cells to the vicinity of the tumor in tissue-based models, are cell-autonomous according to the SMT [6]. As mentioned above, the FBM suggests a cell-heteronomous role for cellular mutations because the function of mutations in loop genes is passive, and the promotion of carcinogenesis by the appropriate tumor microenvironment is necessary. The FBM is also a tissue-based model of carcinogenesis because it assumes that the communication between stromal cells and tumor cells is important for tumorigenesis. However, the FBM is unique from typical tissue-based models because the FBM postulates that the interaction between the different cell types results in a positive feedback loop. Under the FBM, reciprocal activation of the tumor cell and the tumor microenvironment is required for malignant transformation. In the following sections, we will discuss the implications of our FBM on the different pathophysiological features of solid tumors.

### Implications of the Feedback Model on the Relationship Between Inflammation and Cancer

Many cancers are thought to originate from chronic inflammatory environments [40], [41]. Persistent infections with *Helicobacter pylori* cause both gastric cancer and gastric lymphoma. Similarly, hepatitis C infections are strongly associated with hepatocellular carcinomas, and schistosomiasis is a major cause of bladder cancer [12]. The influence of inflammation on carcinogenesis in PDAC has been studied by Guerra *et al.*, who used a mouse model of pancreatic cancer. This study revealed that in adult mice that expressed an exocrine cell-specific somatic KRAS mutation, PanIN lesions and invasive pancreatic cancer did not develop unless chronic pancreatitis was induced [42]. Interestingly, treatment of other mouse models of pancreatic cancer, namely KPC-mice, with anti-inflammatory drugs prolonged the time until the development of tumors [43]. Although KRAS mutations are frequently observed in patients with chronic pancreatitis [44], [45], not every patient with pancreatitis and KRAS mutations develop pancreatic cancer [46]. As postulated above, not every inflammatory microenvironment must inevitably result in a feedback loop even if epithelial cells harbor a mutation in a loop gene.

### Implication on the Tumor Microarchitecture

SMT proposes that cancer cells can proliferate in an unlimited fashion; therefore, one would expect an anarchical distribution of epithelial cells within the tumor. Contrary to this, various solid tumors display defined microarchitectures. The microarchitecture of pancreatic cancer is comparable to chronic pancreatitis [14], [47], [48], which could be explained by the composition of the tumor micromilieu: in our model the tumor microenvironment has a great significance because it comprises the soluble factors that allow the information flow between tumor cells and stromal cells. These factors are comparable to those present during chronic inflammation and are strong chemoattractants for different cell types such as fibroblasts [49]. Indeed, activated pancreatic stellate cells appear to be the most important component of the tumor microenvironment in pancreatic cancer and chronic pancreatitis [48], [50], [51]. However, macrophages and mast cells tend to closely associate with the parenchymal cells as well [24], [25].

Because the signaling molecules involved in the information flow in the FBM are extracellular and soluble, the size of the tumor is important. In very small tumors, the soluble factors within the ECM might easily be affected by changes in the neighboring micromilieu. This change could modify the information flow within the tumor resulting in the termination of the feedback field. After a critical mass of cells becomes adapted to the positive feedback field in larger tumors, the feedback process might be stable enough to be insensitive to changes in the surrounding tissues. The importance of the microenvironment could also explain tumor dormancy, i.e., the variable period of time during which tumor cells exist in a non-proliferative state. Many tumors recur several years after treatment [52]. A previous study has shown that tumor cells are capable of existing as single cell for a prolonged period of time in some organs [53]. Perhaps these dormant tumor cells can be reactivated by a change e.g., in the local microenvironment to one that is inflammatory or otherwise pathological.

### Implications of the Feedback Model on Infiltrative Growth and Metastasis

The capacity of cancer cells to successfully invade and metastasize is dependent on the ability of cancer cells to migrate and to penetrate the ECM. Both of these characteristics are thought to result from the accumulation of additional mutations in other genes [54]. However, several lines of evidence suggest that such mutations are not necessary for metastatic capability. First, migration is accomplished by the downregulation of cell adhesion molecules and the upregulation of cell mobility molecules. The process of acquiring cell migration markers is called epithelial-mesenchymal transition (EMT). EMT is found in cells in different acute and chronic inflammatory conditions [14], [55], [56]. Invasion of epithelial cells in wound healing shows similarities to malignant tumor progression [57]. Therefore, tumor cells may not need to activate EMT by cellular mutations, but EMT could be triggered by the inflammatory microenvironment. Second, infiltration of tumor cells into the extracellular matrix (ECM) is not necessarily dependent on mutations. The intuitive assumption that proteases are produced by the tumor cells has been challenged by the finding that most proteases are derived from stromal cells [58], [59]. Tumor cells might even lack the capacity to actively invade tissue. An electron microscopy study showed that tumor cell invasion into the peritoneum only occurred where the mesothelial cells were damaged and where there was inflammation of the underlying stroma [60].

### Implications of the Feedback Model on Localization of Metastases

To date, the seed-and-soil theory of Paget [61] and the mechanistic theory of Ewing [62] are commonly used to explain the localization of metastases. Compared to the large amount of cells in a typical primary carcinoma, metastasis is a rare event. However, the evaluation of the outcomes of patients with peritoneo-venous shunts for malignant ascites showed that millions of viable tumor cells could be detected in the jugular vein, even though the risk of metastasis was not significantly increased [63].

Among other important molecular mechanisms [59], the assumption of the existence of a positive feedback loop could predict the localization metastases. Cells of the immune system, hormones and cytokines are an integrative part of the tumor microenvironment in the FBM. These components are drained locally by efferent venous circulation or lymphatic vessels [64]. According to our model, primary tumor cells are dependent on these inflammatory cells and factors. Therefore, cancer cells that are separated from the primary tumor will preferentially grow in organs to which the components of the feedback field drain or where inflammatory cells derived from the feedback field localize. Indeed, pancreatic cancer metastasizes preferentially to the lymph nodes and liver [65]. As the burden of tumor cells will eventually grow, the factors of the tumor microenvironment could be traceable within the circulatory system of the patient leading to the development of local and distant metastases.

### Role of Cancer Stem Cells in the FBM

In organs and tissues, normal stem cells reside at the apex of the hierarchal scheme that drives organogenesis. The realization that tumors themselves function as complex organs initiated the concept that cancer cells with the properties of stem cells may be the key drivers of tumorigenesis. This theory has been denoted the cancer stem cell theory [66], [67], which proposes that cancer stem cells arise from genetic mutations amassed by normal adult stem cells. Cancer stem cells in pancreatic cancer are defined by the markers CD44, CD24, ESA and CD133. Pancreatic cancer stem cells are further defined by their potency for tumor formation *in vitro* and *in vivo* [67]. The cancer stem cell theory explains many shortcomings of the SMT. For example, cancer stem cells can survive long enough to accumulate the mutations necessary for malignant transformation [66], [67]. However, the existence of stem cells would not contradict the FBM because stem cells as well as epithelial cells can initiate a feedback loop based on cell signaling aberrancy.

### Possible Models for Investigating the FBM

Models that can directly investigate tissue-based concepts of carcinogenesis are difficult to devise because of the complex nature of these models. Animal models appear to be the most appropriate type of model available for this purpose. Recently, an animal model that selectively expresses endogenous KRAS (G12V) oncogene in centroacinar lineage cells was designed. This mouse could serve as a good model to investigate the effects of different inflammatory conditions on carcinogenesis [42]. Cell lines derived from this mouse model could be used to analyze the effect of co-culture with different cell types on untransformed cells *in vitro*. Still, the investigation of the FBM is limited by the difficulty in analyzing processes involved in early carcinogenesis in humans. Isolation of cell lines from human pancreatic carcinomas with explant culture techniques showed that an initial outgrowth of tumor cells occurred in 51.8% of the tumor samples. However, most of the cell lines senesced, and only 9.25% of the cell lines were capable of being perpetually propagated [68]. These results suggest that the permanent cell lines represent advanced tumors that might have accumulated sufficient mutations to grow *in vitro*. Most permanent cell lines will therefore not represent early stages of carcinogenesis and are not suitable for investigating the FBM.

### Therapeutic Consequences of the Theory

The efficacy of targeting the tumor microenvironment can be found in recent therapeutic strategies for hepatocellular carcinoma. This cancer type is accompanied by a fibrotic stromal reaction consisting of hepatic stellate cells, a physiological response often found in tumor tissues. Recent clinical studies have indicated that chemotherapy for hepatocellular carcinoma could be more effective if therapies targeting the underlying liver fibrosis were also employed [69].

This phenomenon was also shown in pancreatic cancer as well. An experimental approach to reduce fibrosis and, therefore, the inflammatory microenvironment in a mouse model of pancreatic cancer (KPC-mouse) largely enhanced the response towards chemotherapy [70]. Inflammation can also be targeted to reduce the incidence of cancer. Studies on the chemoprevention of cancer showed that chronic suppression of inflammation through the use of non-steroidal anti-inflammatory drugs resulted in a lower incidence of colon and breast cancer [6], [71].

In conclusion, the FBM is unique among the tissue-based models because, in the FBM, the tumor and stromal cells interact to form a positive feedback loop. The result is the perpetuation of an inflammatory/proliferative state of cellular activation. The FBM further postulates that loop genes act in a cell-heteronomous fashion. However, the FBM is thought to supplement, not to supplant the SMT. The present review demonstrates that such a feedback loop is possible, and the FBM model corresponds to many of the characteristics of tumor growth and tumor pathophysiology.

## Materials and Methods

### Literature Search

The protein interactions for Figure 2 were assembled from electronic databases, such as the Kyoto Encyclopedia of Genes and Genomes (www.genome.ad.jp/kegg), Gene Data Base of the National Center for Biotechnology Information (www.ncbi.nlm.nih.gov) and GeneMAPP (www.genmapp.org). For literature search we used PubMed (www.ncbi.nlm.nih.gov/pubmed) and Google Scholar (scholar.google.de).
